# Utility of rituximab treatment for exophthalmos, myxedema, and osteoarthropathy syndrome resistant to corticosteroids due to Graves’ disease: a case report

**DOI:** 10.1186/s13256-018-1571-9

**Published:** 2018-02-16

**Authors:** Aldo Ferreira-Hermosillo, Ruben Casados-V, Pedro Paúl-Gaytán, Victoria Mendoza-Zubieta

**Affiliations:** 1 Unidad de Investigación Médica en Endocrinología Experimental, Hospital de Especialidades, Centro Médico Nacional Siglo XXI, IMSS, Cuauhtémoc 330, colonia Doctores, Delegación Cuauhtémoc, Mexico City, CP 06720 Mexico; 2 Departamento de Endocrinología, Hospital de Especialidades, Centro Médico Nacional Siglo XXI, IMSS, Mexico City, Mexico

**Keywords:** Primary hyperthyroidism, Myxedema, Exophthalmos, Rituximab

## Abstract

**Background:**

Exophthalmos, myxedema, and osteoarthropathy syndrome is a very rare condition that is associated with Graves’ disease. The presence of dermopathy and the involvement of joint/bone tissues indicate that it seems to be related with the severity of the autoimmune process. Owing to its low incidence, there is a lack of information regarding its treatment and clinical follow-up. Some cases improved after use of high doses of steroids; however, some patients do not respond to this treatment. Recently, the effectiveness of rituximab for treatment of Graves’ ophthalmopathy resistant to corticosteroids has been demonstrated. However, it has never been used for the treatment of exophthalmos, myxedema, and osteoarthropathy syndrome (particularly for the treatment of osteoarticular manifestations).

**Case presentation:**

We present the case of a 54-year-old Mexican woman previously treated for Graves’ disease who developed post-iodine hypothyroidism and exophthalmos, myxedema, and osteoarthropathy that did not improve after high doses of steroids (intravenous and oral). Her exophthalmos, myxedema, and osteoarthropathy syndrome symptoms improved as early as 6 months after treatment with rituximab.

**Conclusion:**

Exophthalmos, myxedema, and osteoarthropathy syndrome is a non-classical presentation of Graves’ disease, whose clinical manifestations could improve after treatment with rituximab, particularly in those patients with lack of response to high doses of corticosteroids.

## Background

First reported by Robert James Graves in 1835, Graves’ disease (GD) is characterized by the chronological involvement of thyroid, eye, and skin tissues in long-standing cases [1]. The literature has shown that eye disease, also called Graves’ ophthalmopathy (GO), is not uncommon and can be present not only during thyroid disease, but also years before or after it (even when the patient has normal thyroid function). The prevalence of GO is almost 20 to 40%; and 4 to 13% of patients with GO could develop Graves’ dermopathy. Additionally, 20% of patients with Graves' dermopathy could present acropachy. Rarely other tissues, such as bone or articulations, are affected because of thyrotoxicosis [2].

Exophthalmos, myxedema, and osteoarthropathy (EMO) syndrome is a triad that was first described by Thomas in 1933 [3]. This entity is a very rare condition that affects less than 1% of patients with thyroid disease [4]. Of the few cases reported, 95% were associated with GD and 5% were associated with Hashimoto’s disease. Its etiology remains unknown; but seems to predict the severity of the response of the autoimmune process [5].

We present the case of a patient with GD who developed EMO syndrome. The objective of this case report is to describe this rare extrathyroid manifestation of GD and review current knowledge of its physiopathology and therapeutic options. The severity of the disease requires a multidisciplinary approach that includes an endocrinologist, ophthalmologist, rheumatologist, and a dermatologist.

## Case presentation

A 54-year-old Mexican woman, resident of Mexico City, was referred for evaluation to our Endocrinology department due to severe and painful edema of her legs accompanied by indurate skin tumors, along with palpebral edema and generalized joint pain that caused difficulty in ambulation. She had a family history of gastric cancer (mother) but no history of thyroid disease or other autoimmune diseases. She had a history of appendectomy 15 years ago and GD was diagnosed 2 years ago, which was treated with radioactive iodine (20 mCi of I^131^). She was on thyroid hormone replacement with levothyroxine at time of evaluation. She had no other medical or psychosocial history of relevance.

**Fig. 1:**
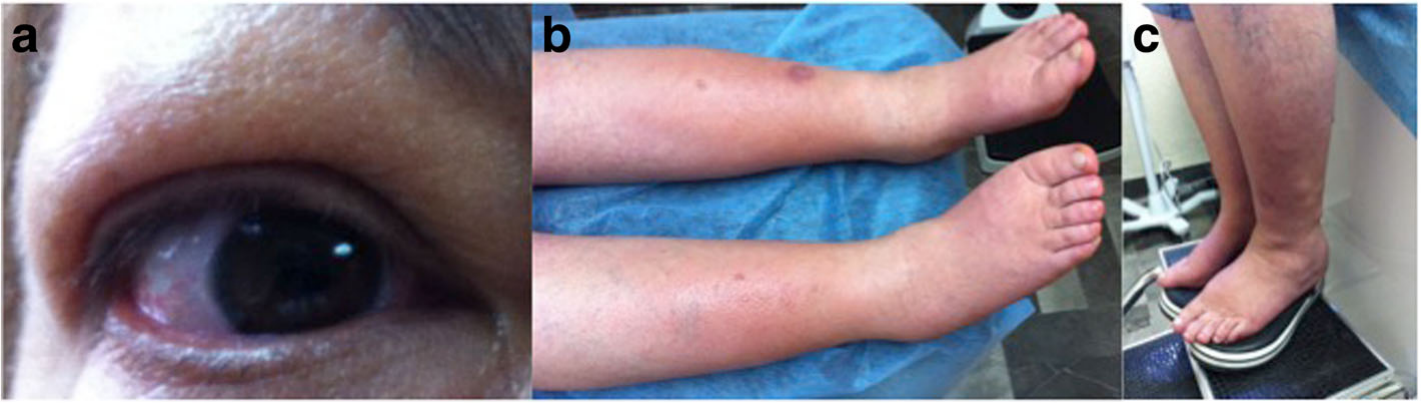
Initial clinical manifestations of the patient with exophthalmos, myxedema, and osteoarthropathy syndrome. Patient arrived with swollen eyelids (**a**), pretibial myxedema with appearance of *peau d’orange* (**b**), and indurated nodules especially notable in her left leg (**c**)

The physical examination revealed bilateral exophthalmos, grittiness, and swollen eyelids leading to mechanical ptosis, and well-demarcated thick erythematous lesions in both legs and feet, indurated and painful on palpation with appearance of *peau d’orange* (pretibial myxedema) and skin nodules of 0.5 cm (Fig. 1). The rest of the examination was normal.

**Fig. 2:**
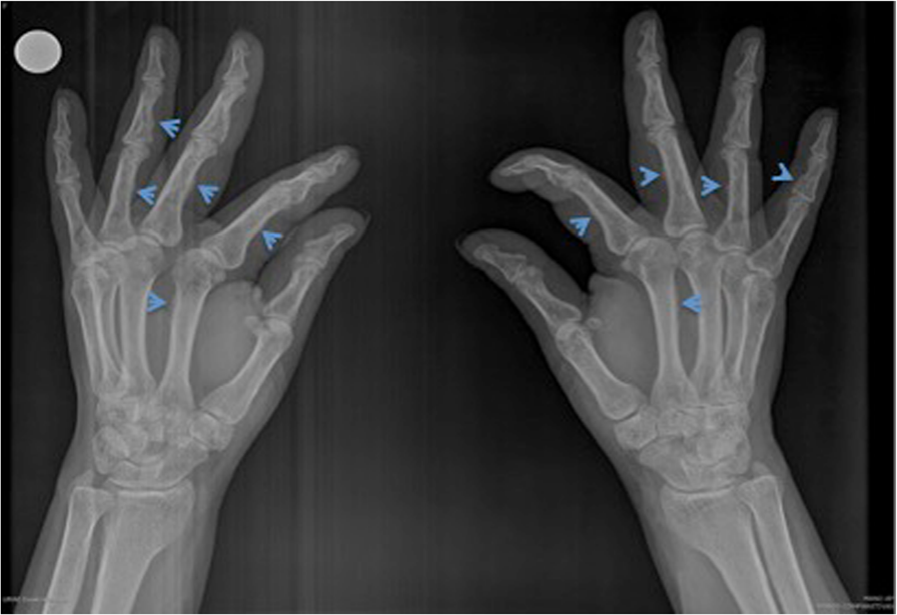
Radiography of our patient’s hands. Radiography showed periostitis of phalangeal and metacarpal bones (*arrowheads*) and joint space narrowing

Laboratory tests reported a normal thyroid-stimulating hormone (TSH) of 2.5 μU/mL (reference 0.27 to 4.2 μU/mL) with free thyroxin (fT4) level of 1.4 ng/dl (0.93 to 1.7 ng/dl), and anti-TSH receptor (anti-sTSHr) antibodies of 49.4 IU/L (range 0 to 1.75 IU/L). Bone dual energy X-ray absorptiometry (DEXA) reported a T-score in her hip of − 1.09, Z-score of 0.44 (osteopenia), and in column a T-score of − 2.82 and Z score of − 1.05 (osteoporosis). Radiographic findings in her hands and feet showed joint space narrowing of the proximal phalanges, carpometacarpal joints, and tarsometacarpal joints, and periostitis, compatible with hypertrophic osteoarthropathy (Fig. 2). Due to the severity of the edema (Fig. 1), it was decided to evaluate skin histopathology of her legs that disclosed pretibial myxedema. In view of her history of thyroid disease and involvement of eyes, skin, and joints, she was diagnosed as having EMO syndrome.

**Fig. 3:**
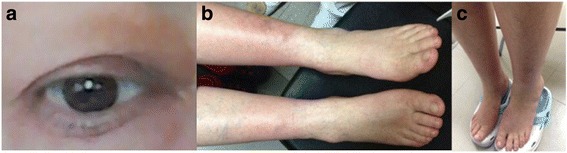
Improve of clinical manifestations after treatment with rituximab. After treatment, there was an obvious improvement in eyelids (**a**), myxedema (**b**), and disappearance of the skin nodules (**c**)

Regarding her treatment, she received methotrexate over a 1-year period with weekly increasing doses up to 15 mg. In addition, she was treated with weekly up-titrating doses of prednisone (15 to 25 mg/day), without clinical improvement. Because of this lack of effectiveness, we decided to suspend methotrexate and prednisone (progressive decrease of 5 mg every week until definitive suspension) and prescribed rituximab after an adequate evaluation discarding viral infections such as human immunodeficiency virus (HIV), hepatitis B and C, bacterial and fungal infections (through blood culture), and tuberculosis. She also had a normal blood count and hypo/agammaglobulinemia was ruled out (as indicated by our Rheumatology department). Six months after addition of rituximab (two doses of 500 mg by intravenous injection 2 weeks apart), there was a significant clinical improvement. Eight months after treatment, myxedema and nodules disappeared (Fig. 3) and she recovered her leg movement capacity without pain. During her last evaluation a year after treatment, slight exophthalmos still persisted, but there were no clinical signs of eye inflammation, she could close her eyelids without difficulty, and her eye movements are preserved.

## Discussion

EMO syndrome is a rare entity characterized by an intense autoimmune response. Its pathophysiology remains unknown, but it seems to be caused by autoantibodies against thyroid antigens, specifically TSH receptor autoantibodies (TSHr-Ab) and reactive T-lymphocytes that develop a cross-reaction with connective tissue and muscle antigens. In the connective tissue, TSHr-Ab seem to stimulate fibroblasts to produce an increased amount of glycosaminoglycans (GAGs), which also promote liquid retention, separation of collagen fibers, and finally expansion of connective tissues [4, 5].

GO is the most common extrathyroidal manifestation of GD, and typically follows a biphasic course. High serum levels of TSHr-Ab correlate positively with its clinical features, constitute an independent risk factor, and predict the severity and progression of the disease [6]. Approximately 3 to 7% of patients with GO had a severe sight-threatening form with corneal exposure or compressive optic neuropathy [7]. In this report, our patient had a moderate to severe form of ophthalmopathy. Treatment depends on each patient’s characteristics and ranges from lubricating or topical treatment, use of antioxidants, use of steroidal treatment and, in severe cases, immunomodulatory therapy, orbital decompression, eyelid surgery, or even corneal grafting is required [8]. However, as seen in this case, 20 to 25% of patients do not respond to immunosuppressive treatment [9].

The pathogenesis of dermopathy and acropachy also involve the lymphocyte-derived cytokine activation of fibroblasts in the locations involved, similar to that of ophthalmopathy. These dermatologic conditions are associated with GO and its presence usually indicates a more severe autoimmune disease [10]. Graves’ dermopathy usually affects the pretibial area but may occur at any place exposed to repetitive trauma or pressure, such as in the face, arms, shoulders, abdomen, pinna, and previous scars. A typical lesion is a diffuse, non-pitting edema with “orange skin” appearance. Other forms include plaques and nodular lesions, and the most severe is the elephantiasic form [11]. A biopsy of those lesions shows the presence of GAGs and damage in collagen and elastin fibers [12]. Regarding treatment, topical corticosteroids (with additional occlusive dressings) are usually recommended [13]; however, some lesions can be resistant to this type of treatment and in those cases the intralesional application of steroids has been used. At this point, octreotide injections have also been successful in some cases [14, 15].

Thyroidal acropachy presents with digital clubbing, digital and toes swelling, and exuberant periosteal proliferation, located mainly at the small tubular bones of the hands and feet. It is nearly always associated with exophthalmos and pretibial myxedema [16]. Radiological features in patients with thyroid acropachy include development of a characteristic subperiosteal spiculated, frothy, or lacy appearance of periosteal reaction. Nowadays, the only treatment available for this condition is a systemic immunosuppressive therapy and local corticosteroid therapy [17].

Treatment of EMO syndrome as a special entity is difficult and there is a lack of information regarding treatment strategies when high doses of steroids fail. In our case, our patient did not improve despite treatment with steroids and methotrexate. We prescribed rituximab in order to improve eye disease as previously reported and observed that it also improved her skin and joints manifestations. Rituximab is a monoclonal antibody directed to CD20 on B-lymphocytes and it has been proposed to remove those cells in their progression to plasma cells, which consequently prevents their production of autoantibodies as TSHr-Ab [18]. There are 49 cases of thyroid disease treated with rituximab in the medical literature [19]. Précausta *et al*., who observed that rituximab is effective to treat corticosteroid-resistant GO with optic neuropathy, reported the last six cases [20]. In contrast, there are few case reports of rituximab use in thyroid dermopathy. In Australia, a woman treated with rituximab and plasmapheresis improved her movement range in fingers and ankles, and recovered the capacity to ambulate and perform activities of daily living [18]. A 56-year-old man was started on a combination with intravenously administered immunoglobulin and rituximab and improved his disease progression [21].

## Conclusions

Although treatment with rituximab is costly and patients require close clinical evaluation prior to its application (particularly the absence of co-infections), it is undoubtedly a successful treatment in patients with EMO syndrome that did not respond to steroid treatment. Due to the scarcity of information on therapeutic strategies in patients with EMO syndrome, we decided to publish our experience with the use of this drug.

## Abbreviations

anti-sTSHr: Anti-thyroid-stimulating hormone receptor
DEXA: Dual energy X-ray absorptiometry
EMO: Exophthalmos, myxedema, and osteoarthropathy
fT4: Free thyroxin
GAGs: Glycosaminoglycans
GD: Graves’ disease
GO: Graves’ ophthalmopathy
TSH: Thyroid-stimulating hormone
TSHr-Ab: Thyroid-stimulating hormone receptor autoantibodies
